# Mild MPP^+^ exposure-induced glucose starvation enhances autophagosome synthesis and impairs its degradation

**DOI:** 10.1038/srep46668

**Published:** 2017-04-26

**Authors:** Shuichiro Sakamoto, Masatsugu Miyara, Seigo Sanoh, Shigeru Ohta, Yaichiro Kotake

**Affiliations:** 1 Graduate School of Biomedical and Health Sciences, Hiroshima University, Hiroshima, 734-8553, Japan; 2 Global Career Design Center, Hiroshima University, Hiroshima, 739-8514, Japan

## Abstract

Parkinson’s disease (PD) is a prevalent neurodegenerative disorder, mainly characterised by the progressive loss of dopaminergic neurons. MPP^+^ has been widely used as a PD-related neurotoxin, and their reports suggested the several hypotheses for neuronal cell death. However, most of these hypotheses come from the studies about the acute MPP^+^ exposure. We previously revealed that mild MPP^+^ exposure (10 and 200 μM), which induces gradual cell death, impairs autophagosome degradation at 48 h. In the present study, we further investigated the specific events of mild MPP^+^ exposure and revealed that mild MPP^+^ exposure causes the cell death through glucose starvation, but not acute toxic model (2.5 and 5 mM). At 36 h after mild MPP^+^ exposure, autophagosome synthesis was enhanced owing to glucose starvation and continued to enhance until 48 h, despite impaired autophagosome degradation. Inhibition of autophagosome synthesis reduced mild MPP^+^-induced cell death. In conclusion, we clarified that glucose starvation-enhanced autophagosome synthesis occurs at an earlier stage than impaired autophagosome degradation and is important in mild MPP^+^ toxicity.

Parkinson’s disease (PD) is the second most common neurodegenerative movement disorder characterised by resting tremor, slowness of movement, postural instability and muscle rigidity. The most prominent pathological features of PD are the loss of dopaminergic neurons in the *substantia nigra pars compacta* and the appearance of protein inclusions, known as Lewy bodies.

1-Methyl-4-phenyl-1,2,3,6-tetrahydropyridine (MPTP), known to induce irreversible motor abnormalities in humans and primates, has been extensively studied as an etiologic model of PD. The neurotoxic effects of MPTP are attributable to its metabolite 1-methyl-4-phenylpyridinium (MPP^+^), which is formed by monoamine oxidase B-mediated oxidation^1^,^2^. Several MPTP administration protocols have been proposed to produce mouse models of PD, including acute, sub-chronic and chronic treatments^3^. In acute and sub-chronic models, dopaminergic neurons have been shown to die quickly, with little progression in the loss of nigrostriatal dopamine. Furthermore, inclusion bodies are not found in the remaining dopaminergic neurons^3^. Several chronic MPTP mouse models have been developed to replicate the slow progression of PD and have exhibited slower decreases in dopaminergic cell numbers as well as the formation of inclusion bodies^3^,^4^,^5^,^6^. Therefore, a chronic MPTP administration protocol would more suitably replicate the nature of PD.

Many cell-based studies on the mechanism underlying MPP^+^ toxicity have suggested several hypotheses for neuronal cell death, including respiration deficit, energy failure, calcium homeostasis dysregulation, excitatory input, oxidative stress and apoptosis execution^7^. However, most studies supporting these hypotheses performed with acute MPP^+^ exposure to induce adverse effects within 24 h; therefore, the toxicity of mild MPP^+^ exposure remains controversial. In our previous report, we developed a mild MPP^+^ exposure model that might more appropriately replicate PD pathology^8^. In brief, SH-SY5Y human neuroblastoma cells were exposed to low concentrations of MPP^+^ (10 and 200 μM) for 48 h. Exposure to 10 μM MPP^+^ for 48 h induced the accumulation of α-synuclein (α-syn), a major component of Lewy bodies. In the present study, we discovered that mild MPP^+^ exposure induced glucose starvation, leading to cell death, whereas acute MPP^+^ exposure (2.5 and 5 mM for 24 h) did not. Therefore, we investigated the mechanism underlying the specific toxicity of mild MPP^+^.

## Results

### The toxic effects of mild MPP^+^ exposure are distinct compared with those of acute MPP^+^ exposure

**Figure 1:**
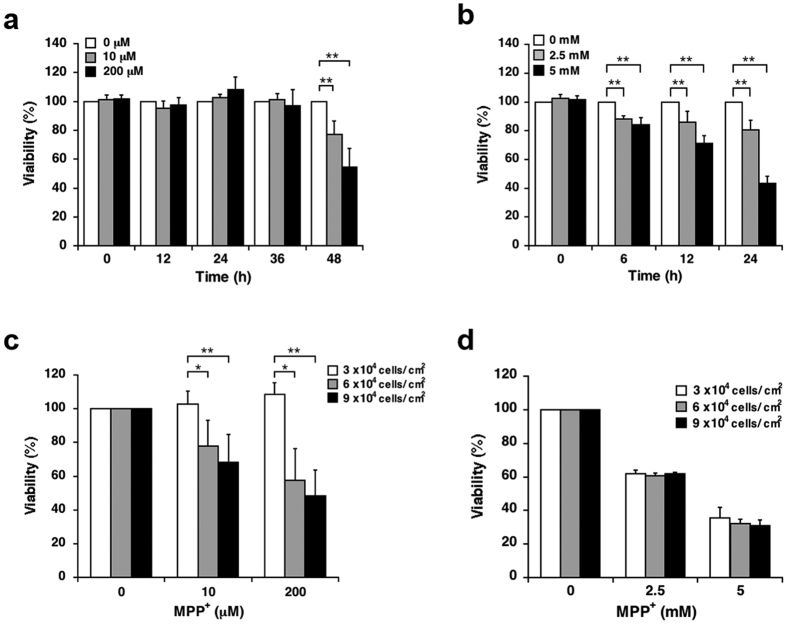
Differences in the toxic effects of mild and acute MPP^+^ exposure. (**a**,**b**) SH-SY5Y cells were exposed to MPP^+^ for up to 48 h or to 2.5 and 5 mM MPP^+^ for up to 24 h; cell viability at various time points was determined using the WST-1 assay. (**c**,**d**) Various densities of SH-SY5Y cells were exposed to 10 and 200 μM MPP^+^ for 48 h or 2.5 and 5 mM MPP^+^ for 24 h, and cell viability was determined using the WST-1 assay. Data are expressed as means ± standard deviations (S.D.) from at least three independent experiments. *p < 0.05, **p < 0.01.

In our previous report, we used 10 and 200 μM MPP^+^ for mild exposure models and 2.5 and 5 mM MPP^+^ for acute toxic exposure models^8^. To validate the utility of the former, we further evaluated differences between the two models. SH-SY5Y were exposed to predetermined concentrations of MPP^+^ for up to 24 h or 48 h, after which cell viabilities were evaluated using the WST-1 assay. A time course analysis revealed that exposure to 10 and 200 μM MPP^+^ significantly decreased cell viability after 48 h but not within 36 h (Fig. 1a), whereas exposure to 2.5 and 5 mM MPP^+^ decreased cell viability within 6 h in a manner that appeared to be time dependent (Fig. 1b).

We further sought to elucidate differences in toxicity between the two models; the toxicity in the mild MPP^+^ exposure model significantly increased in a cell density-dependent manner, whereas that in the acute toxic exposure model did not (Fig. 1c,d).

### Mild MPP^+^, but not acute toxic, exposure induces glucose starvation, leading to cell death

**Figure 2:**
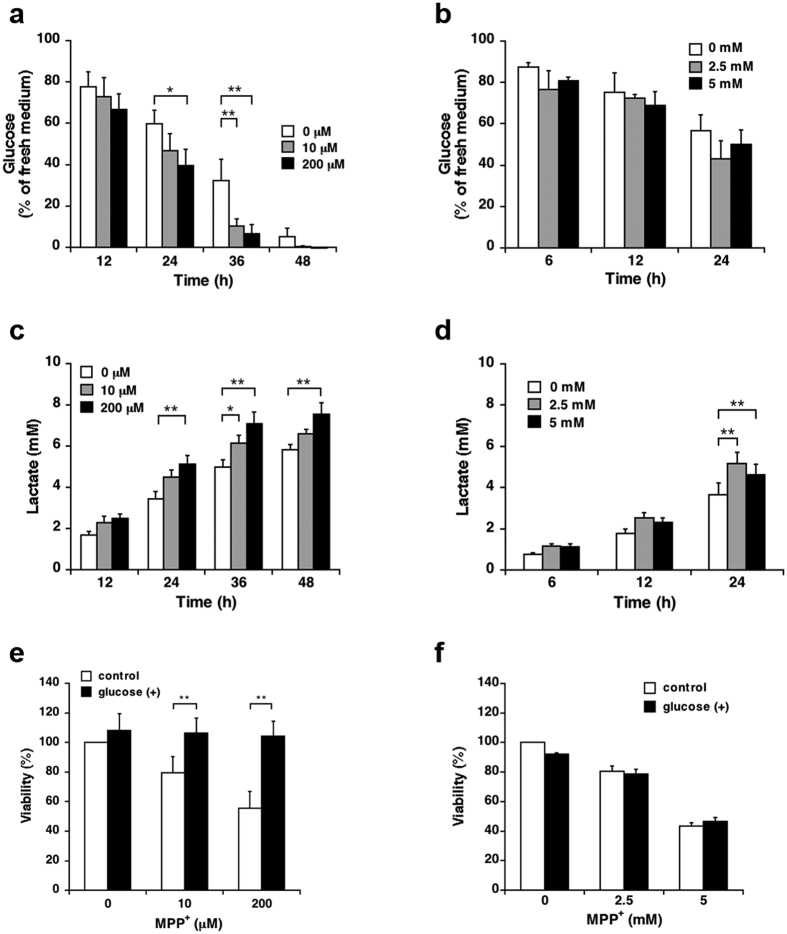
Differences in energy metabolism between cells subjected to mild or acute MPP^+^ exposure. (**a**,**b**) SH-SY5Y cells were exposed to 10 and 200 μM MPP^+^ for up to 48 h or 2.5 and 5 mM MPP^+^ for up to 24 h; glucose concentrations in the culture media at several time points were measured using a commercial assay kit. (**c**,**d**) SH-SY5Y cells were exposed to 10 and 200 μM MPP^+^ for up to 48 h or 2.5 and 5 mM MPP^+^ for up to 24 h; extracellular lactate concentrations at several time points were measured using a commercial assay kit. (**e**,**f**) SH-SY5Y cells were exposed to 10 and 200 μM MPP^+^ for 48 h or 2.5 and 5 mM MPP^+^ for 24 h in the presence of 5.5 mM glucose for the last 12 h. Cell viability was determined using the WST-1 assay. Data are expressed as means ± S.D. from at least three independent experiments. *p < 0.05, **p < 0.01.

Because a higher cell density expedited cell death only under mild MPP^+^ exposure, we hypothesised that the difference in toxicity between the mild and acute MPP^+^ exposure models stemmed from the rate of consumption of a particular nutrient in the culture medium. MPP^+^ inhibits mitochondrial oxidative phosphorylation by inhibiting complex I of the mitochondrial electron transport chain (ETC)^9^, thereby facilitating anaerobic glycolysis^10^. Therefore, we evaluated the effects of mild and acute MPP^+^ exposures on glycolytic activity. First, we measured the amount of glucose in the culture medium; the exposure to 10 and 200 μM MPP^+^ resulted in significant reductions in extracellular glucose levels within 36 and 24 h, respectively (Fig. 2a), whereas the exposure to 2.5 and 5 mM MPP^+^ did not result in significant decreases within 24 h (Fig. 2b). We next determined the amount of extracellular lactate, a product of glycolytic metabolism. During exposure to 10 and 200 μM MPP^+^, significant increases in extracellular lactate were evident within 36 and 24 h, respectively (Fig. 2c). These results correlated with the extracellular glucose analysis results. In contrast, the model of acute MPP^+^ exposure also led to a significant increase in extracellular lactate within 24 h; however, this result did not correlate with the extracellular glucose analysis results (Fig. 2b,d).

We further investigated the effect of glucose supplementation on toxicity resulting from mild and acute MPP^+^ exposures. In brief, SH-SY5Y cells were exposed to each concentration of MPP^+^ and supplemented with 5.5 mM (1 g/L) glucose for the last 12 h. Cell death consequent to mild MPP^+^ exposure was significantly reduced by glucose supplementation (Fig. 2e). In contrast, no significant differences were detected in the model of acute MPP^+^ exposure (Fig. 2f).

### Mild MPP^+^-induced glucose starvation facilitates autophagosome accumulation

**Figure 3:**
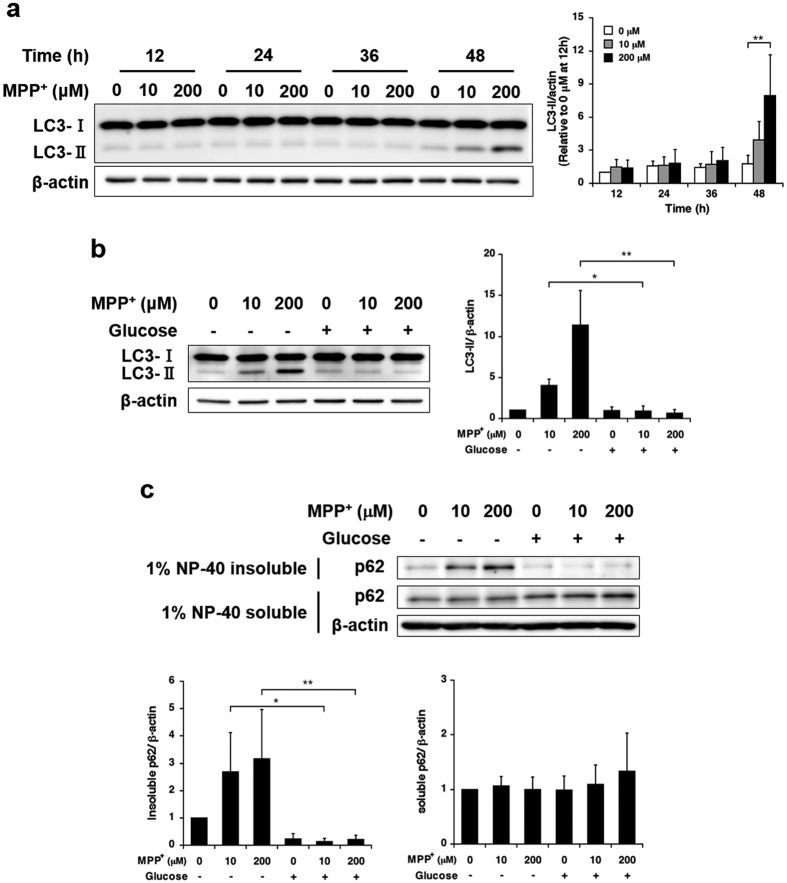
Influence of glucose supplementation on mild MPP^+^ exposure-induced autophagosome accumulation. (**a**) SH-SY5Y cells were exposed to 10 and 200 μM MPP^+^ for up to 48 h; LC3-II expression levels at several time points were detected by western blotting. (**b**) SH-SY5Y cells were exposed to 10 and 200 μM MPP^+^ for 48 h with or without 5.5 mM glucose for the last 12 h; LC3-II expression was detected by western blotting. (**c**) SH-SY5Y cells were exposed to 10 and 200 μM MPP^+^ for 48 h with or without 5.5 mM glucose for the last 12 h; p62 expression was detected by western blotting. Data are expressed as means ± S.D. from at least three independent experiments. *p < 0.05, **p < 0.01.

Autophagy is a lysosomal degradative process through which cytosolic constituents and damaged organelles are recycled. Related substrates are sequestered in double-membraned vesicles called autophagosomes, which then fuse with lysosomes^11^. We previously reported that mild MPP^+^ exposure induces autophagosome accumulation within 48 h as a result of impaired autophagosome degradation^8^. Therefore, we assessed the relationship between glucose starvation and autophagosome accumulation during mild MPP^+^ exposure. Western blot analysis revealed that exposure to 10 and 200 μM MPP^+^ significantly increased the expression of LC3-II, an autophagosomal marker, within 48 h but not within 36 h (Fig. 3a), and glucose supplementation significantly suppressed the increase in the expression of LC3-II (Fig. 3b). Our previous report showed that mild MPP^+^ exposure induced the accumulation of p62/SQSTM1 (p62), a selective autophagy substrate, in a 1% Nonidet P-40 (NP-40) insoluble fraction of lysed cells^8^, and we would further expect this substrate to be sequestered and aggregated in non-degraded autophagosomes. Furthermore, the accumulation of p62 in this insoluble fraction was also suppressed by glucose supplementation (Fig. 3c).

### Mild MPP^+^ exposure enhances autophagosome synthesis

**Figure 4:**
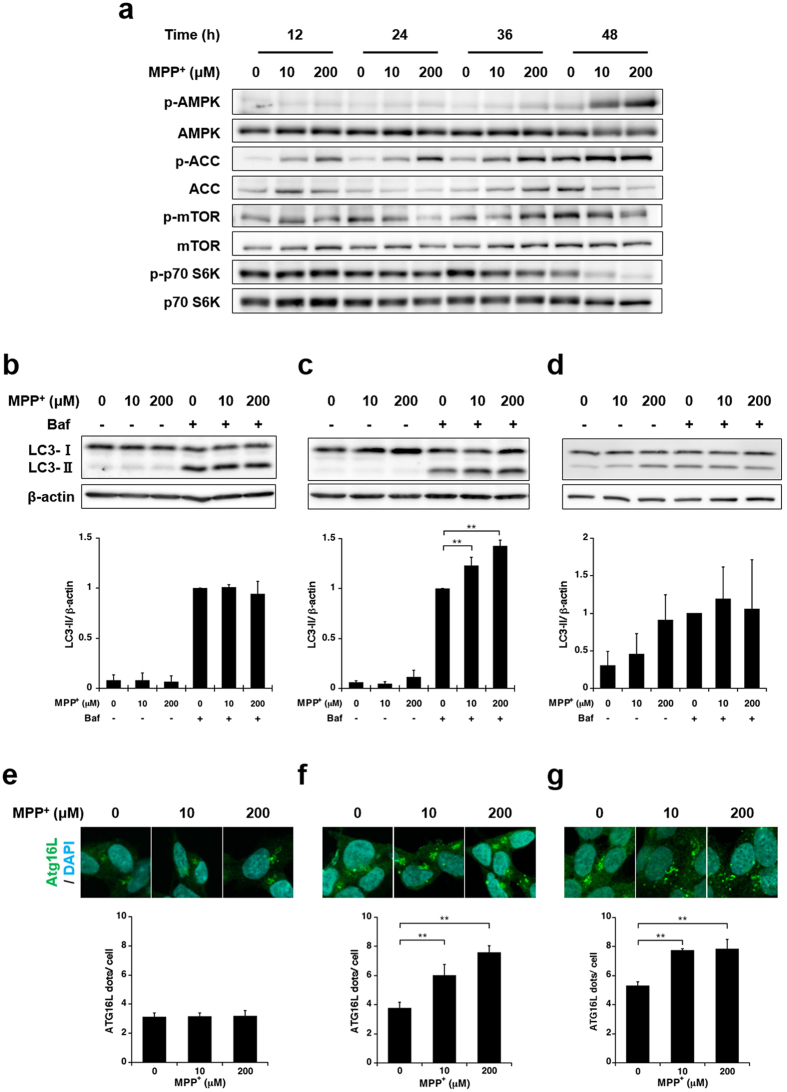
Time-dependent changes in autophagic induction with mild MPP^+^ exposure. (**a**) SH-SY5Y cells were exposed to 10 and 200 μM MPP^+^ for up to 48 h. Time-dependent changes in the phosphorylation levels of various proteins were estimated by western blotting. (**b**,**c**,**d**) SH-SY5Y cells were exposed to 10 and 200 μM MPP^+^ for 24 h (**b**), 36 h (**c**) or 48 h (**d**) with or without 200 nM bafilomycin A_1_ (Baf) for the last 4 h; LC3-II turnover was estimated by western blotting at various time points. (**e**,**f**,**g**) SH-SY5Y cells were exposed to 10 and 200 μM MPP^+^ for 24 h (**e**), 36 h (**f**) or 48 h (**g**) and subsequently immunostained with an anti-Atg16L antibody. The numbers of Atg16L-positive puncta per cell were evaluated at various time points. Data are expressed as means ± S.D. from at least three independent experiments. **p < 0.01.

Various signalling events can enhance autophagy; of these, nutrient starvation (e.g. depletion of amino acids, growth factors, or glucose) is among the most effective enhancers of autophagosome synthesis^12^. Starvation-related signalling pathways converge at the mammalian target of rapamycin (mTOR), a central protein kinase of the nutrient-sensing pathway^13^. Conditions of starvation enhance autophagy and thus increase energy levels by recycling cellular components, thereby increasing the possibility that mild MPP^+^-induced glucose starvation enhances autophagosome synthesis. Under glucose starvation, adenosine monophosphate (AMP)-activated protein kinase (AMPK) is activated by an increase in the AMP/adenosine triphosphate (ATP) ratio; this leads to the inactivation of mTOR, a negative regulator of autophagy, and thus induces autophagy^14^. Western blot analysis revealed that exposure to 10 and 200 μM MPP^+^ for 48 h increased the phosphorylation of AMPK, and consequently, its substrate acetyl-CoA carboxylase (ACC) but decreased the phosphorylation of mTOR and its target, p70 S6K. In addition, increases in AMPK and ACC phosphorylation were already detected within 12 h, whereas reductions in mTOR and p70 S6K phosphorylation were apparent 36 h onward (Figs 4a and S1a). Similar to LC3-II expression levels, these changes were suppressed by glucose supplementation (Fig. S1b).

To investigate enhanced autophagosome synthesis, we evaluated the turnover of LC3-II. In brief, SH-SY5Y cells were exposed to 10 or 200 μM MPP^+^ with or without 200 nM bafilomycin A_1_ (Baf) for the last 4 h of exposure, after which LC3-II protein expression was estimated by western blotting. Baf is a specific inhibitor of vacuolar-type H^+^-ATPase that prevents fusion between autophagosomes and lysosomes, thus preventing the lysosomal degradation of LC3-II. Because changes in the phosphorylation of ACC and p70 S6K were confirmed within 48 h, we investigated the turnover of LC3-II at various time points (24, 36 and 48 h). At 24 h, neither 10 nor 200 μM MPP^+^ exposure increased the levels of LC3-II expression, regardless of Baf treatment (Fig. 4b). However, exposure to 10 and 200 μM MPP^+^ did not increase LC3-II expression in the absence of Baf at 36 h, although both concentrations increased the levels of LC3-II expression in the presence of Baf (Fig. 4c). At 48 h, exposure to 10 and 200 μM MPP^+^ increased LC3-II expression in the absence of Baf, whereas neither concentration increased LC3-II expression in the presence of Baf (Fig. 4d). These results suggest that mild MPP^+^ exposure enhances autophagosome synthesis within 36 h but inhibits autophagosome synthesis and autophagosome degradation within 48 h.

To further determine whether mild MPP^+^ exposure enhances autophagosome formation, we measured the numbers of Atg16L positive puncta at various time points via immunocytochemical analysis. During the autophagic process, LC3-I, the cytosolic form of LC3, is conjugated to the membrane lipid phosphatidylethanolamine and converted to an autophagosome membrane-bound form (LC3-II)^15^. The Atg12–Atg5–Atg16L1 complex, which only exists in the autophagosomal precursor membrane, is necessary for the conjugation of LC3-I to phosphatidylethanolamine^16^,^17^.

Therefore, an increase in Atg16L-positive puncta is indicative of enhanced autophagosome biogenesis^18^. At 24 h, we did not observe a significant increase in Atg16L-positive puncta in response to 10 and 200 μM MPP^+^ (Fig. 4e). In contrast, exposure to 10 and 200 μM MPP^+^ resulted in a significant increase in Atg16L-positive puncta at 36 h; this result, which was similar to the LC3-II turnover findings, suggested enhanced autophagosome synthesis (Fig. 4c,f). At 48 h, although LC3-II turnover suggested that autophagosome synthesis is not enhanced, exposure to 10 and 200 μM MPP^+^ led to significant increases in Atg16L-positive puncta (Fig. 4g). In the MPP^+^-untreated group, Atg16L-positive puncta increased in a time-dependent manner (Fig. 4e–g). Furthermore, a decrease in p70 S6K phosphorylation was evident in the MPP^+^-untreated group at 36 h and exacerbated at 48 h (Fig. 4a). These results suggest enhanced autophagosome synthesis in the MPP^+^-untreated group from 36 h to 48 h. Because the LC3-II turnover assay incorporates comparative evaluation between the MPP^+^-untreated group and the treated group in the presence of Baf, it might not precisely distinguish autophagosome synthesis in response to mild MPP^+^ exposure at 48 h. Collectively, our results suggest that mild MPP^+^ exposure enhances autophagosome synthesis after 36 h.

### Enhanced autophagosome synthesis contributes to toxicity associated with mild MPP^+^ exposure

**Figure 5:**
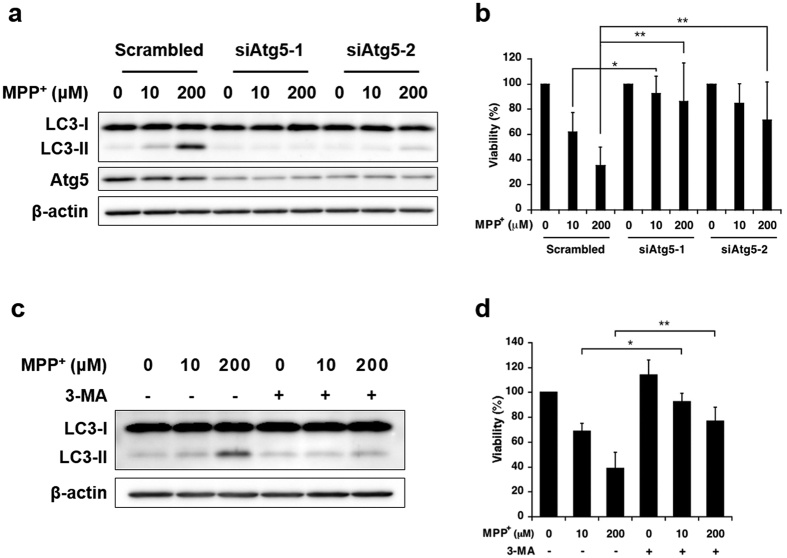
Influence of autophagy inhibition on toxicity associated with mild MPP^+^ exposure. (**a**) SH-SY5Y cells were transfected with siRNA specific for autophagy protein 5 (siAtg5) or scrambled control siRNA for 24 h and subsequently exposed to 10 and 200 μM MPP^+^ for 48 h. LC3-II and Atg5 expression levels were analysed by western blotting. (**b**) SH-SY5Y cells were transfected with siAtg5 or scrambled control siRNA for 24 h, followed by exposure to 10 and 200 μM MPP^+^ for 48 h. Cell viability was determined by a WST-1 assay. (**c**) SH-SY5Y cells were exposed to 10 and 200 μM MPP^+^ for 48 h with or without 5 mM 3-MA for the last 24 h; LC3-II expression was analysed by western blotting. (**d**) SH-SY5Y cells were exposed to 10 and 200 μM MPP^+^ for 48 h with or without 5 mM 3-MA for the last 24 h; cell viability was determined by the WST-1 assay. Data are expressed as the means ± S.D. from at least three independent experiments.

We assessed the relationship between enhanced autophagosome synthesis and toxicity associated with mild MPP^+^ exposure. Small interfering RNA (siRNA)-mediated knockdown of autophagy protein 5 (Atg5), an essential component of LC3 conjugation, effectively inhibited the increase in LC3-II levels in SH-SY5Y cells exposed to 10 or 200 μM MPP^+^ for 48 h (Fig. 5a). Furthermore, the toxicity associated with exposure to 10 and 200 μM MPP^+^ for 48 h was partially reduced by Atg5 knockdown (Fig. 5b).

3-Methyladenine (3-MA), a widely used autophagy inhibitor, inhibits class III PI3K activity, an essential component in the induction of autophagosome synthesis. Notably, because 3-MA is usually used at very high concentrations for autophagy inhibition, other kinases and cellular processes may be affected^19^,^20^,^21^. To minimise these effects, SH-SY5Y cells were exposed to 10 and 200 μM MPP^+^ with or without 5 mM 3-MA for only the last 24 h. 3-MA also inhibited the increase in LC3-II and attenuated the toxicity associated with 10 and 200 μM MPP^+^ exposure (Fig. 5c,d). Moreover, autophagy inhibition did not significantly increase the amount of glucose in the culture medium (Fig. S2). Taken together, our data suggest that enhanced autophagosome synthesis is involved in the toxicity associated with mild MPP^+^ exposure.

## Discussion

MPP^+^ is selectively assimilated by dopaminergic neurons and inhibits complex I of the mitochondrial ETC^9^. It is reported that MPP^+^ toxicity occurs simultaneously with reductions in phosphocreatine and ATP as well as increases in lactic acid and glucose utilisation in different cultures, including neuroblastoma, primary neuron and rat brain slice^10^,^22^,^23^,^24^. In addition, supplementation of glucose, a major energy source for the brain, has been reported to prevent MPP^+^ toxicity and ATP reduction by increasing anaerobic glycolysis activity^25^,^26^,^27^,^28^. In the present study, we revealed that mild MPP^+^ exposure induced glucose starvation and led to cell death, whereas acute MPP^+^ exposure led to glucose starvation-independent cell death. During mild MPP^+^ exposure, we observed the onset of cell death with simultaneous glucose depletion (Figs 1a and 2a). Moreover, cell death was significantly suppressed by glucose supplementation, even at 36 h after mild MPP^+^ exposure (Fig. 2e). These results imply that mild MPP^+^ exposure induces cell death through glucose starvation and the subsequent lack of energy.

We previously reported that exposure to 10 μM MPP^+^ induced the accumulation of α-syn within 48 h^8^, and glucose starvation was also reported to induce the accumulation of α-syn in mouse primary mesencephalic neurons^29^. Both enhanced glycolytic activity and accumulation of α-syn in the brain were confirmed in a chronic MPTP mouse model produced using a continuous minipump infusion system^30^. Thus, mild MPP^+^ exposure may reflect the chronic MPTP mouse model. It is also reported that mutation of PD-related genes which relate to quality control of mitochondria induce enhanced glycolytic activity and accumulation of α-syn. Parkin-mutant *Drosophila* larvae exhibited defective locomotion with a bradykinesia-like phenotype and showed reduced oxygen and ATP concentrations and an increased lactate concentration^31^. Parkin knockdown and knockout activates glycolysis and reduces mitochondrial respiration in cultured cells^32^. Glycolysis is also enhanced by the knockout of two other PD-related genes, PTEN-induced putative kinase 1 (PINK1) and DJ-1^33^,^34^,^35^,^36^. Moreover, both PINK1 and DJ-1 associated with the accumulation of α-syn^37^,^38^.

Acute MPP^+^ exposure did not significantly alter the amount of glucose in the culture medium within 24 h (Fig. 2b). If acute MPP^+^ exposure does not facilitate the glucose consumption, a reduction in the amount of glucose in the culture medium progresses more slowly than that in the amount of glucose in the MPP^+^-untreated group because of the reduced number of living cells. Therefore, these data implicate that acute MPP^+^ exposure enhances glycolytic activity (Figs 1a and 2b). However, cell death occurred even when sufficient glucose was retained in the culture medium (Figs 1b and 2b). Moreover, glucose supplementation did not alleviate acute MPP^+^-induced cell death (Fig. 2f). These results suggest that the model of acute MPP^+^ exposure induces many events, other than glucose starvation, that are essential for cell death. Although the amount of glucose in the medium did not significantly decrease within 24 h of acute MPP^+^ exposure, extracellular lactate levels significantly increased within the same period (Fig. 2b,d). α-Ketoglutarate dehydrogenase (α-KGDH), an enzyme in the tricarboxylic acid (TCA) cycle, is also inhibited by MPP^+^ in a concentration-dependent manner^39^,^40^. Therefore, acute MPP^+^ exposure might strongly suppress TCA cycle activity through α-KGDH inhibition, leading to an increase in extracellular lactate levels.

The presence of Lewy bodies or abnormal protein aggregates has been confirmed in patients with PD, dementia with Lewy bodies and other diseases, and both LC3 and p62 have been identified in Lewy bodies from patients’ brains^41^,^42^,^43^. These reports imply a relationship between abnormal autophagy and Lewy body formation. Several groups have reported that MPP^+^ exposure increases the number of autophagosomes in cell cultures. However, consensus has yet to be reached regarding the proposed mechanism underlying this phenomenon because some studies suggest enhanced autophagosome synthesis^44^,^45^,^46^,^47^ and others report impaired autophagosome degradation^48^,^49^. This discrepancy implies that the effects of MPP^+^ exposure on the autophagic process vary depending on the exposure condition and/or cell type. In the present study, we revealed glucose starvation to be the cause of impaired autophagosome degradation during mild MPP^+^ exposure (Fig. 3). We further suggest that mild MPP^+^ exposure enhances autophagosome synthesis via glucose starvation (Figs 4 and S1). Although we did not detect an increase in LC3-II expression levels in SH-SY5Y cells exposed to mild MPP^+^ within 36 h (Fig. 3a), our results shown in Fig. 4 suggested that autophagosome synthesis was already enhanced at 36 h. SH-SY5Y cells were previously found to exhibit minimal increases in LC3-II after nutrient deprivation, despite enhanced autophagosome synthesis, whereas treatment with a lysosomal inhibitor or autophagosome fusion inhibitor facilitates the detection of increased levels of LC3-II^50^,^51^, likely because of high basal autophagic flux in cells of neuronal origin^52^,^53^. Mature neurons cannot dissipate intracellular protein aggregates through cell division; therefore, autophagic quality control of intracellular proteins is crucial for neuronal survival^54^. Indeed, brain-specific autophagy-deficient mice exhibited protein aggregate formation, neuronal cell death and neurodegenerative disorder-like symptoms^55^,^56^. However, our data showed that the suppression of autophagosome synthesis alleviated mild MPP^+^-induced cell death. Inhibition of autophagosome synthesis did not significantly affect the cellular glucose consumption (Fig. S2). Therefore, Enhanced synthesis under the conditions of impaired autophagosome degradation might facilitate autophagosome accumulation, thus disrupting intracellular protein quality control. Zhu *et al*. investigated the toxicity associated with chronic MPP^+^ exposure and suggested the presence of enhanced autophagosome synthesis via extracellular signal-regulated protein kinases (ERK)^57^. Moreover, these authors suggested that the suppression of autophagosome synthesis reduced cell death. Because glucose starvation increases the levels of reactive oxygen, thereby enhancing autophagosome synthesis via p38 mitogen-activated protein kinases, c-Jun N-terminal protein kinase, or ERK^14^, our mild MPP^+^ exposure model also likely upregulates ERK through glucose starvation. On the other hand, Zhu *et al*. confirmed the presence of enhanced autophagosome synthesis but not impaired autophagosome degradation^57^. Therefore, both phenomena might have occurred in their chronic model.

In summary, mild MPP^+^ exposure induced cell death through glucose starvation, accelerated non-degraded autophagosome accumulation correlates with cell death and toxicity associated with mild MPP^+^ exposure differs from that associated with acute MPP^+^ exposure. The present study might reveal the relationship between abnormal glucose metabolism and dysfunctional autophagic machinery in PD.

## Methods

### Chemicals

MPP^+^ iodide and 3-Methladenine were purchased from Sigma-Aldrich, D-(+)-Glucose from Wako, Bafilomycin A_1_ from Cayman chemical.

### Cell cultutre

Human neuroblastoma SH-SY5Y cells were purchased from American Type Culture Collection (ATCC, CRL-2266). The cells were grown in Dulbecco’s modified eagle medium (low-glucose) supplemented with 0.854 g/L L-glutamine, 5% fetal bovine serum, 5% horse serum, and 0.2% sodium hydrogen carbonate under a humidified atmosphere of 95% air-5% CO_2_ at 37 °C. For experiments, cells were seeded at a density of 6.04 × 10^4^ cells/174 μL/cm^2^ and incubated overnight. The medium was changed just before MPP^+^ treatment in all experiments.

### Cell viability analysis

Cell viability was measured with the water-soluble tetrazolium salt (WST)-1 assay. The cells were seeded in 96-well plate, incubated overnight. After treatment, cells were incubated for 1 h at 37 °C with a mixed solution of WST-1, 1-methoxy-5-methylphenazinium methylsulfate, and DMEM. The absorbance of the converted dye was measured at 415 nm using a MultiSkan Go microplate spectrophotometer (Thermo Fisher Scientific).

### Glucose assay

Glucose in the culture medium was evaluated using the Glucose colorimetric/fluorometric assay kit (Bio Vision, K606-100) according to the manufacture’s instructions. Briefly, cells were seeded in a 24-well plate, incubated overnight, and treated with MPP^+^. The culture medium were collected and then centrifuged at 400× g for 5 min at 4 °C. Aliquot of supernatant from each samples was added to the reaction solution and incubated for 30 min at 37 °C. The absorbance at 570 nm was measured with a plate reader.

### Glycolytic activity assay

Glycolytic activity was determined by measuring the amount of L-lactate, the end product of glycolysis. The activity was measured using glycolysis cell-based assay kit (Cayman chemical, 600450) according to the product manual. Briefly, cells were seeded in a 96-well plate, incubated overnight, and treated with MPP^+^. The culture medium were collected and then centrifuged at 400× g for 5 min at 4 °C. Aliquot of supernatant from each samples was added to the reaction solution and incubated with gentle shaking on an orbital shaker for 30 min. The absorbance at 490 nm was measured with a plate reader.

### 1% NP-40-soluble and –insoluble fraction

The cells were seeded in 60-mm dish, incubated overnight, and treated with MPP^+^. After treatment cells were washed three times with PBS(−), then lysed with TNE buffer containing 1% Nonidet P-40 [20 mM Tris-HCl, pH 7.4, 150 mM NaCl, 2 mM EDTA, 1% Nonidet P-40, 1 mM sodium fluoride, 1 mM sodium orthovanadate, 1% protease inhibitor cocktail]. The cell lysates were sonicated on ice, rotated for 30 min at 4 °C, and centrifuged at 22,000× g for 20 min at 4 °C. The supernatants were collected as 1% Nonidet P-40-soluble fractions. Then, the pellets were further lysed in TNE buffer containing 2% SDS [20 mM Tris-HCl, pH 7.4, 150 mM NaCl, 2 mM EDTA, 1% SDS, 1 mM sodium fluoride, 1 mM sodium orthovanadate, 1% protease inhibitor cocktail], sonicated on ice, rotated for 30 min at room temperature (RT), and collected as 1% Nonidet P-40-insoluble fractions. Protein concentration of each sample extract was determined with Pierce BCA Protein Assay Kit (Thermo Fisher Scientific, 23227). Cell lysates were heated with Laemmli’s sample buffer at 95 °C for 5 min.

### Immunoblot analysis

Equivalent amounts of protein (5–20 μg) from each sample were loaded and separated on SDS-PAGE, then transferred to a PVDF membrane. Membranes were blocked with 5% skim milk in TBS supplemented with 0.1% Tween 20 (TBS-T) for 1 h at RT. The membranes were probed with anti-LC3 anti body (MBL, PM036), anti-p62/SQSTM1 antibody (MBL, M162-3), anti-phospho-AMPK α (Thr172) (Cell Signaling, #2535), anti-AMPK α (Cell Signaling, #2532), anti-phospho-ACC (Ser79) (Cell signaling, #3661), anti-ACC (Cell Signaling, #3662), anti-phospho-mTOR (Ser2448) (Cell signaling, #5536), anti-mTOR (Cell Signaling, #2972), anti-phospho-p70 S6K (T421/S424) (R&D Systems, AF8965), anti-p70 S6K (R&D Systems, AF8962), anti-Atg5 (MBL, PM050), or anti-β-actin antibody (Sigma-Aldrich, A5441) diluted in 5% skim milk or Can Get Signal^®^ Immunoreaction Enhancer Solution (TOYOBO) for 2 h at RT or overnight at 4 °C. After washing with TBS-T three times, membranes were probed with HRP-conjugated goat anti-rabbit IgG antibody (Sigma-Aldrich, A9169) or HRP-conjugated anti-mouse IgG antibody (Sigma-Aldrich, A9044) for 1 h at RT. After washing with TBS-T three times, membranes were incubated Chemi-Lumi One L (Nacalai Tesque) or Chemi-Lumi One Ultra (Nacalai Tesque). Specific bands were detected using a luminescent image analyzer (GE Healthcare, ImageQuant LAS 4000); relative band intensities were quantified by densitometric analysis using Image J.

### Immunocytochemical analyses

SH-SY5Y cells were seeded in poly-d-lysine-coated four-well chamber slides (BD Biosciences, 354577) and incubated overnight at 37 °C in a humidified 5% CO_2_ incubator. Cells were exposed to MPP^+^ as indicated, washed with PBS(−), and fixed with 4% paraformaldehyde in PBS(−) for 10 min at RT. Fixed cells were washed with PBS(−), permeabilized twice with PBS(−) containing 0.1% triton X-100 (PBS-Tx) for 15 min at RT, blocked with 3% BSA in PBS-Tx for 1 h, and incubated with a rabbit anti-Atg16L antibody (MBL, PM040) diluted in PBS-Tx for 2 h for RT. Labeled cells were then washed three times with PBS-Tx, incubated with Alexa Fluor 488-conjugated goat anti-rabbit IgG (Life Technologies, A11008) diluted in PBS-Tx for 1 h at RT in the dark, washed three times with PBS(−), incubated with 600 nM DAPI (Life Technologies, D1306) in PBS(−) for 5 min at RT in the dark, washed twice in PBS(−), and mounted in Prolong Diamond antifade reagent (Life Technologies, P36961). After incubation overnight, slides were observed under a confocal laser scanning microscope (Olympus, FV1000-D IX81). Images were processed using ImageJ software. Graph: Average number of Atg16L-positive puncta per cell. At least n = 10 fields with at least 100 cells per condition from three independent experiments.

### RNA interference

Silencing of Atg5 was performed with siAtg5 (Ambion, s18158 and s18160) or scrambled control (Ambion, AM4611). Transfection of siRNAs (10 nM) was performed using Lipofectamine RNAiMAX Transfection Reagent (Thermo Fisher Scientific) according to the manufacture’s protocol. 24 h after transfection, cells were transferred into the appropriate plate or dish for experiments.

### Statistical analysis

Data are expressed as the mean ± standard deviations (S.D.) from at least three independent experiments. Significant differences between multiple independent groups were determined by Tukey-Kramer test with probability value (*p*) < 0.05 considered significant.

## Additional Information

**How to cite this article**: Sakamoto, S. *et al*. Mild MPP^+^ exposure-induced glucose starvation enhances autophagosome synthesis and impairs its degradation. *Sci. Rep.* **7**, 46668; doi: 10.1038/srep46668 (2017).

**Publisher's note:** Springer Nature remains neutral with regard to jurisdictional claims in published maps and institutional affiliations.

## Supplementary Material

Supplementary Figures
