# Supplementary material for: Mild MPP+ exposure-induced glucose starvation enhances autophagosome synthesis and impairs its degradation
Source: Sci Rep. 2017 Apr 26;7:46668. doi: 10.1038/srep46668 (PMC5405408; doi:10.1038/srep46668)
Supplement: Supplementary Figures [file srep46668-s1.pdf]

Supplementary figures

Mild MPP$^+$ exposure-induced glucose starvation enhances autophagosome synthesis and impairs its degradation.

Shuichiro Sakamoto[1], Masatsugu Miyara[1,2], Seigo Sanoh[1], Shigeru Ohta[1] & Yaichiro Kotake[1,*]

[1]Graduate School of Biomedical and Health Sciences, Hiroshima University, Hiroshima, 734-8553, Japan

[2]Global Career Design Center, Hiroshima University, Hiroshima,739-8514, Japan

* To whom correspondence should be addressed: Tel: +81-82-257-5326; E-mail: yaichiro@hiroshima-u.ac.jp

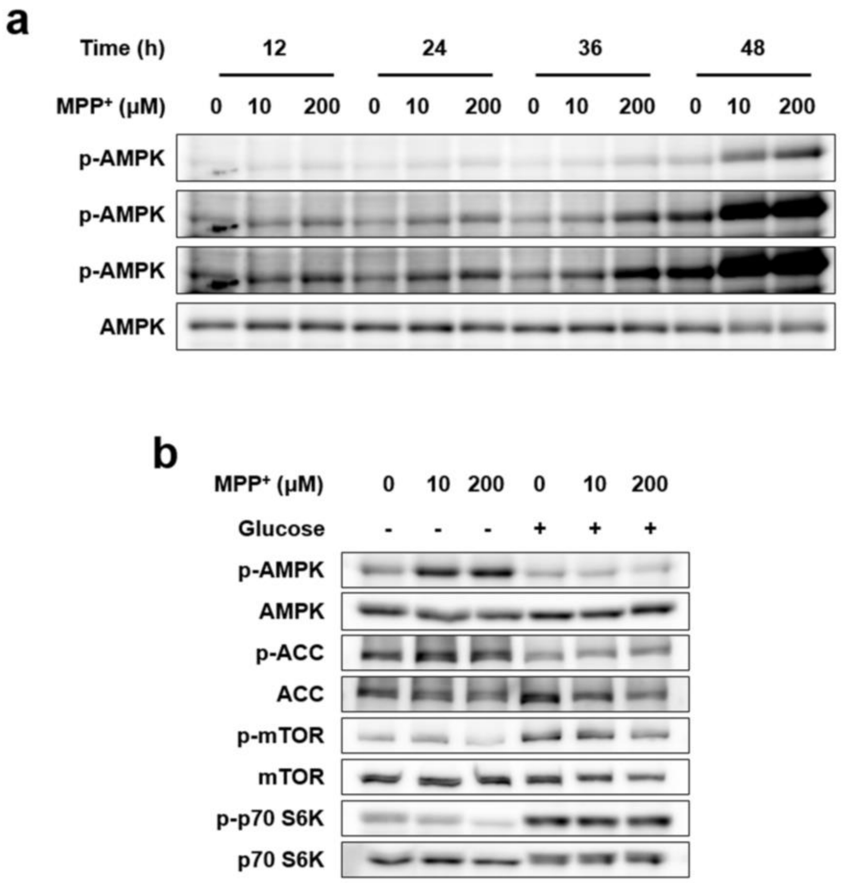

Supplementary figure 1. Influence of mild MPP$^+$ exposure on glucose starvation-induced autophagic signal. (a) SH-SY5Y cells were exposed to 10 and 200 μM 1-methyl-4-phenylpyridinium (MPP$^+$) for up to 48 h; p-AMP-activated protein kinase (AMPK) expression at several time points was detected by western blotting. Upper bands were clearly detected in groups exposed to MPP$^+$ for 48 h. Middle p-AMPK bands were clearly detected in groups exposed to MPP$^+$ for 36 h. Lower p-AMPK bands were clearly detected in groups exposed to MPP$^+$ for 12 and 24 h. (b) SH-SY5Y cells were exposed to 10 and 200 μM MPP$^+$ for 48 h with or without 5.5 mM glucose for the last 12 h. The phosphorylation levels of various proteins were estimated by western blotting.

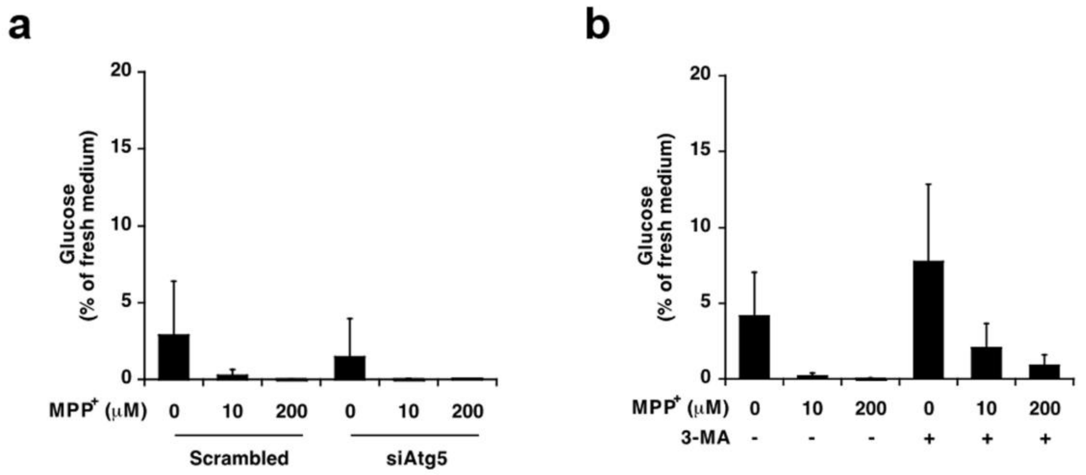

Supplementary figure 2. Influence of autophagy inhibition on glucose consumption associated with mild MPP$^+$ exposure. (a) SH-SY5Y cells were transfected with siRNA specific for autophagy protein 5 (siAtg5) or scrambled control siRNA for 24 h and subsequently exposed to 10 and 200 μM MPP$^+$ for 48 h; glucose concentrations in the culture media were measured using a commercial assay kit. (b) SH-SY5Y cells were exposed to 10 and 200 μM MPP$^+$ for 48 h with or without 5 mM 3-MA for the last 24 h; glucose concentrations in the culture media were measured using a commercial assay kit. Data are expressed as the means ± S.D. from at least three independent experiments.
